# Microplastics in the Water Column of the Rhine River Near Basel: 22 Months of Sampling

**DOI:** 10.1021/acs.est.3c08364

**Published:** 2024-03-13

**Authors:** Gabriel Erni-Cassola, Reto Dolf, Patricia Burkhardt-Holm

**Affiliations:** † Man-Society-Environment (Programme MGU), Department of Environmental Sciences, University of Basel, Vesalgasse 1, Basel CH-4051, Switzerland; ‡ Abteilung Umweltlabor, Amt für Umwelt und Energie, Department für Wirtschaft, Soziales und Umwelt des Kantons Basel-Stadt, Spiegelgasse 15, Basel CH-4001, Switzerland

**Keywords:** microplastics, discharge, Rhine monitoring station, suspended particulate matter

## Abstract

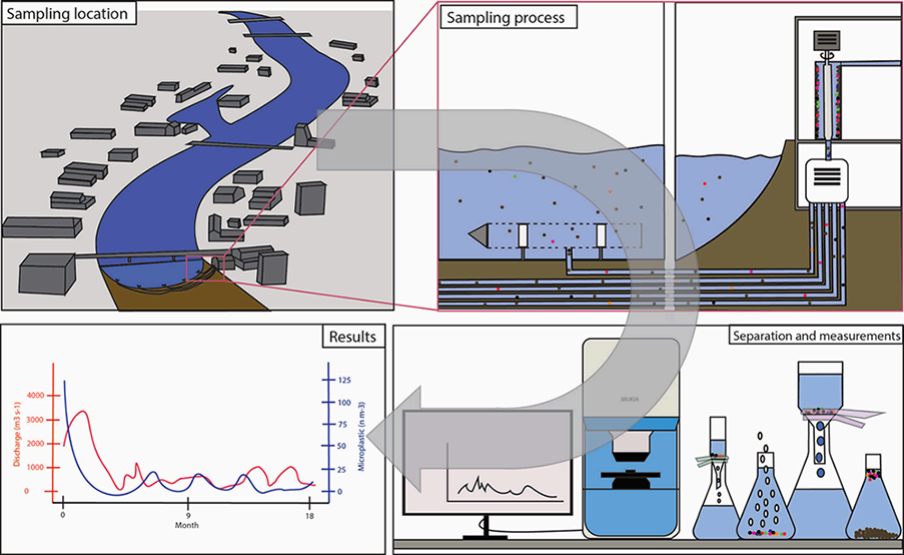

Measured microplastic concentrations in river surface waters fluctuate greatly. This variability is affected by season and is codriven by factors, such as sampling methodologies, sampling site, or sampling position within site. Unfortunately, most studies comprise single-instance measurements, whereas extended sampling periods are better suited to assessing the relevance of such factors. Moreover, microplastic concentrations in riverine water column remain underexplored. Similar to the oceans, however, this compartment likely holds significant amounts of microplastics. By representatively sampling the entire Rhine River cross-section near Basel through five sampling points over 22 months, we found a median microplastic (50–3000 μm) concentration of 4.48 *n* m^–3^, and estimated a widely ranging load between 4.04 × 10^2^*n* s^–1^ and 3.57 × 10^5^*n* s^–1^. We also show that the microplastic concentration in the water column was not well explained by river discharge. This suggests that although high discharge events as observed here can over short time periods lead to peak microplastic concentrations (e.g., 1.23 × 10^2^*n* m^–3^), microplastic load variance was not dominated by discharge in the study area.

## Abstract 2

Over 22 months the Rhine River exported microplastics in the water column in a chemodynamic pattern, which informs environmental modeling.

## Introduction

Environmental plastic pollution is growing, as projections indicate that by 2050 about 12,000 million metric tons of plastics will have been discarded in landfills and the natural environment, compared to today’s 4900 t.^1^ In the natural world, small plastic fragments (microplastics; 1–5000 μm) move in water and air and deposit even in remote areas.^2−4^ Although microplastics share properties, such as the size range, density range, or longevity, with other naturally occurring particles like clay minerals or particulate organic matter, the combination of these properties in microplastics makes them distinct particles,^5^ with uncertain negative impacts on exposed biota.^6−10^ Although the global plastic flux between the different environmental pools has received considerable research attention, important knowledge gaps remain.^11^

Rivers can be viewed as key conveyor belts for aquatic plastic pollution, and based on mismanaged plastic waste, estimates suggest that up to 2.7 t are delivered to oceans yearly.^12^ Although more recent studies using population and drainage intensity yielded lower estimates (6.1 kt year^–1^^13^), riverine plastic emissions are still projected to account for 12–13% of the plastic input into the oceans.^14^ Models and field studies highlight that the input is subject to seasonal variability, such as increased contributions during the monsoon period, or heightened discharges in Europe, between February and April.^15,16^ Yet, a majority of the studies investigating plastic pollution entail single-instance assessments, which highlight consistent plastic pollution, albeit varying by up to 7 orders of magnitude.^16,17^ Variability is codriven by factors, such as differing sampling methodology, sampling site, and sampling position within site.^16^ The Rhine River for instance, has been widely investigated for plastic pollution in surface waters along the profile from Basel to Rotterdam,^18^ its shorelines in the Mainz area,^19^ benthic midstream sediments in the Lower Rhine,^20^ and wastewater treatment plant effluents and suspended particulate matter in the Netherlands.^21^ Although seasonal effects have been hypothesized to explain some of the observed variability, a study designed to assess seasonal patterns did not find clear effects.^22^ Nonetheless, in other river systems precipitation has been found to drive microplastic concentrations in surface water,^23,24^ stressing the recognized need for more studies with higher time-resolution in freshwaters.^16,25,26^ These data are needed to assess the contribution of river water column microplastics to the ocean water column, which is an important transitory sink of microplastics in the ocean.^27^

To improve our understanding of microplastic transport dynamics in rivers, it is necessary to not only study particles transported at the surface, but to also consider further layers of fluvial transport, such as particles suspended in the water column, i.e., suspended and wash loads.^28^ Elucidating suspended loads is important to help test model assumptions when estimating global microplastic river transport, by informing how to extrapolate from surface loads to wash loads.^28,29^ In a marine setting, recent studies of plastic pollution in and below the mixed layer (e.g.^30,31^) have contributed toward improved mass budget estimations of marine plastics revealing that 36–39% reside in the deeper ocean.^14^ By contrast, data on suspended microplastic loads in rivers remain particularly scarce.^16,26,32^

The aim of the present study was to conduct a time-resolved study of the microplastic concentration in the wash load of the Rhine River water column near Basel. Given that microplastic concentrations can differ starkly depending on the streamwise location of the sampling site,^16,18^ we aimed to sample across the entire river cross section. For the latter, we relied on protocols established for the standardized monitoring program of the International Commission for the Protection of the Rhine (ICPR), of which assessing microplastic concentrations is not currently a part of. We hypothesized that the relative abundance of buoyant polymer types is lower in the wash load compared to samples from surface waters in the same region and tested whether river discharge correlates with microplastic number concentrations in the water column.

## Materials and Methods

### Sampling Location

Rhine River water column was sampled in the Basel area at the Rhine Monitoring Station in Weil am Rhein (Rhine km 171.37; 47° 36′ 4.8528″, 7° 35′ 35.2314″). The sampling site is situated in the impounded section of the river, which starts at the outflow of Lake Konstanz and ends at the dam of Iffezheim.^33^ At the site, the river is 200 m wide, and reaches a depth of 10 m; the mean bed slope of the impounded region is 1 m km^–1^.^33^ While there is no gravel transport, clay, silt, and to a lesser extent also sand, are transported through the impounded section with the most significant input upstream of Basel, i.e., the Aare River.^33^ Although regulated by dams, the discharge regime is snowmelt determined. The impounded Rhine section is open to commercial shipping starting in Basel with its port upstream of the sampling site (Rhine km 169–170). As the Rhine River in the study area drains, ca. 68% of Switzerland, additional potential sources of microplastics may be highly diverse. The catchment area of the River in Basel corresponds to 36,400 km^2^, with a mean discharge of 1050 m^3^ s^–1^. Most of the catchment area (55%) is forested or otherwise kept near-natural, 38% is agricultural land, 4% are water areas, and built-up area constitutes 4%.^34^

### Sampling Procedure

**Figure 1:**
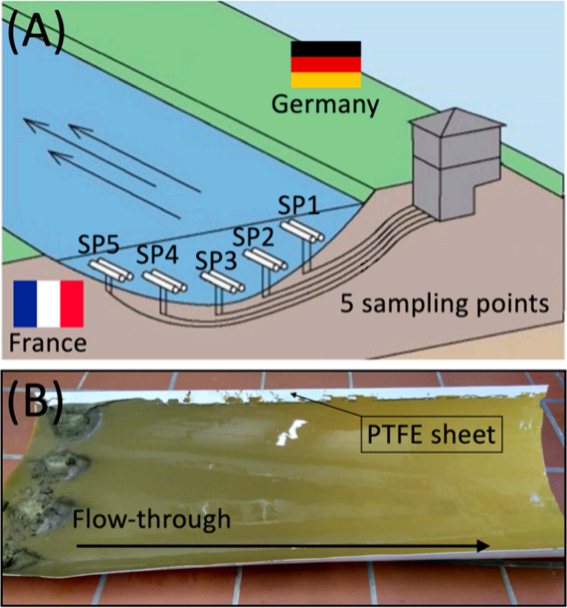
Schematic illustration of Rhine Monitoring Station in Weil am Rhein (Germany) sampling points (SP) in river bed (A) and example of PTFE collection sheet with suspended particulate matter (B). (A) modified from Amt für Umwelt and Energie (AUE), Kanton Basel-Stadt.^73^

Samples of suspended particulate matter (SPM) for microplastic analysis were collected monthly starting in July 2021 through to March 2023. These consisted of opportunistic samples, obtained through a long-running, standardized monitoring program in the context of the International Commission for the Protection of the Rhine (ICPR). SPM is sampled via five sampling points installed in the riverbed at a depth ranging from 3.7 to 8 m (Figure 1A; Table 1). By covering the entire cross-section, the setup is designed to enable representative sampling of the entire river width. Water was drawn via stainless steel pipes and led into a flow-through centrifuge (61G, type CEPA Carl Padberg Zentrifugenbau GmbH, Germany), where SPM was collected on a polytetrafluoroethylene (PTFE) sheet (17,000 rpm, 21,000 G). The diameter of the centrifuge inflow was 3 mm, which determined the expected size maximum of potential microplastics. Centrifuge run time was determined by water turbidity with the aim of collecting 100–300 g of suspended particulate matter by wet weight and set to prevent overloading the PTFE collection sheet; the maximum run-time per sampling event was 5 days. Therefore, sampling durations differed accordingly (12–98 h, median = 91.8 h), sampling between 2.23 m^3^ and 22.5 m^3^ of water (median = 19.6 m^3^). The wet weight of the total collected SPM ranged between 78.9–527 g (median = 246 g; for details see Table S1).

**Table 1:** Summary of Water Column Sampler Set-Up; See fig. 1a for Schematic Overview

Sampling Point	Distance to Station [m]	Sampling Depth [m]	River Depth [m]	Fraction of Mixture^1^
SP1	34	5	6	0.05
SP2	56	8	10	0.17
SP3	104	7.5	9	0.30
SP4	132	5.5	7.6	0.25
SP5	182	3.7	4.8	0.23

1 Mixture represents Rhine River cross section at the Rhine Monitoring Station Weil am Rhein; ratio was experimentally determined for the standardized long-term monitoring program of the International Commission for the Protection of the Rhine (ICPR).

For microplastics analysis, the collected SPM was subsampled by scraping a fraction off the PTFE sheet with a wooden spatula (width ca. 6 cm), thereby aiming to collect from end to end, against the flow through direction (Figure 1B) at least 20 g by wet weight. Due to the opportunistic nature of the study, it was, however, not always possible to meet the latter aim (Table S1). The subsampled fraction constituted between 5 and 25% (median = 13%) of the total by wet weight (Table S1), and represented a median of 1.39 m^3^ water, ranging from 0.1 to 3.86 m^3^. The SPM samples were immediately transferred to a glass container and closed with a metallic lid for transport.

### Sample Preparation

To extract potential microplastics, a protocol inspired by Mani et al.^20^ was used, relying on oxidative sample digestion via Fenton’s reaction followed by density separation and overflow using saturated NaBr solution.^35^ Samples were dried using a vacuum drier at 60 °C for 72 h. For digestion 10 g of sample were transferred into 250 mL Erlenmeyer flasks. Then 15 mL FeSO_4_ (20 mg mL^–1^, pH = 3) were added and the flasks placed in a water bath at room temperature. Ensuite, 15 mL of H_2_O_2_ (30%) were added dropwise over the course of 15 min followed by sonication for 5 min at 160 W/35 kHz; the latter two steps were repeated once. Fenton’s reaction was left to conclude overnight before drying the samples in a vacuum drier at 60 °C. To perform the density separation, NaBr solution (ρ = 1.5 g cm^–3^) was added to the flasks until about 2 cm below the brim. The samples were sonicated for 5 min and stirred for 5 min to ensure that all of the material had been resuspended. Samples were then left for 24 h to sediment. For the overflow, flasks were placed in glass Petri dishes (14 cm diameter) and topped up with NaBr solution injecting about 2 cm below the surface, taking care to minimize resuspension; flasks were left for another 30 min. Floating material was then decanted through a glass funnel into a collection bottle. To improve extraction rates, density separation steps were repeated once.^36^

The collected supernatant was vacuum filtered through a 500 μm mesh (PTFE, 47 mm diameter) to collect large particles, while small particles were retained on a 20 μm stainless steel mesh (47 mm diameter; Wolftechnik Filtersysteme GmbH & Co.KG, Germany). Collected material was copiously rinsed with ultrapure water to remove NaBr. To remove ferric oxide, HCl (5%) was additionally pipetted onto the samples. Large particles (>500 μm) were stored at least 3 days on the filter mesh in a glass Petri dish until further analysis. Particles retained on the 20 μm stainless steel mesh were rinsed off with ultrapure water, and collected on an aluminum oxide filter membrane (13 mm diameter, 0.2 μm pore size; Whatman Anodisc); filters were stored in a dry cabinet for a minimum of 24 h.

To assess the particle size distribution in a subset of SPM samples with sufficient leftover material, laser diffraction with 3 min ultrasound energy for aggregate dispersion was used (Malvern Mastersizer 2000 with a Hydro 2000, Malvern Instruments Ltd.).

### Particle Characterization and Spectroscopy

Particles in a size range of 50–3000 μm were analyzed. For each sample, all large particles (>500 μm) were first photographed under a binocular microscope (Olympus SZ61, 45× magnifying, camera: Olympus SC50), and measured at their largest cross-section using Olympus CellSens software, as described previously;^22^ the longest axis was defined as major length, and the axis perpendicular to it as minor length. To then assess the chemical identity of suspected microplastics, attenuated total reflection Fourier-transform infrared spectroscopy (ATR FTIR) was performed. Each particle was placed on the crystal and compressed to record a spectrum in the range of 4000–400 cm^–1^ with a resolution of 4 cm^–1^, and a total of 24 coadded scans (model Alpha, Bruker Optics GmbH., Billerica, MA, USA).

To assess the chemical identity of the small particles (50–500 μm), an FT-IR microscope was used (model Lumos, Bruker Optics GmbH., Billerica, MA, USA). In the controlling software (OPUS, Bruker Optics GmbH), each filter was photographed, and all the particles with a major length >50 μm were selected for automated scanning following;^37^ all others were excluded from the analysis. Particles were scanned in transmission mode with an aperture of 50 × 50 μm between 4000 and 1200 cm^–1^, at a resolution of 4 cm^–1^, and a total of 64 coadded scans. Data were extracted from the OPUS file into single spectrum CSV files using a custom script in MATLAB (R2021a Update 4), retaining data in the range 3300 < *x* > 1300 wavenumber cm^–1^. The particles confirmed as synthetic polymers (see *Data treatment and statistical analysis*) were measured at their largest cross-section in OPUS. As detailed characterization of specific shapes was not systematically recorded for the purposes of this study, the term ’particle’ is used as a general descriptor for microplastic materials identified in the samples.

### Data Treatment and Statistical Analysis

Data processing and library searching was performed using functions provided in the R package *OpenSpecy*.^38^ For library searches using raw data, spectra were smoothed and baselines corrected using default parameters, i.e., the Savitzky–Golay algorithm (polynomial degree 3, filter length 11), and IModPolyFit (polynomial degree 8). Processed spectra were then used to search for matching entries in libraries provided by the package (Chabuka and Kalivas;^39^ Primpke et al.;^40^ Thermo Fisher Scientific). In parallel, library searches were conducted using the first derivative of the spectra; the latter were calculated using the Savitzky–Golay algorithm, also applying smoothing (polynomial degree 3, filter length 11). Derivatives emphasize peak positions and can lead to better search results.^41,42^ For a library match to be selected, a Pearson correlation coefficient >0.7 was adopted.^43^ As additional check, each selected match to a synthetic polymer was visually assessed, and accepted matches grouped into clusters following.^40^

Mass of small microplastic particles was estimated in line with previous studies.^44,45^ The ratio of the minor length to major length (see *Particle characterization and spectroscopy*) was calculated for each particle, yielding a median value of 0.63. Assuming that the ratio of the thickness to the minor dimension of a particle was equivalent, thickness was estimated as 63% of the minor dimension.^45^ Individual particle masses were then inferred by calculating the volume of an ellipsoid shape as the best “one shape fits all” approximation and multiplying by the density of the corresponding polymer type; used densities are listed in Table S2.

Plastic particle number and mass estimates were converted into number and mass concentrations based on mean Rhine River discharge during each sampling event. This conversion was done in conjunction with the flowthrough centrifuge runtime (see Table S1) and the total dry weight of suspended particulate matter. Microplastic monthly load (*L*) was estimated based on measured concentration, and the average Rhine River discharge in the area at the respective sampling event, assuming a homogeneous distribution of particles. To compare the fractions of different polymer types over the entire sampling period, weighted mean fractions were used and 95% confidence intervals estimated via bootstrapping; resampling (*n* = 10,000) was performed using the package boot.^46^ To explore the factors influencing microplastic number concentrations in the water column, linear models were employed. These models used as explanatory variables average discharge (*Q*) during each sampling event and suspended particulate matter concentration. The response variable was the microplastic number concentration. Analogously, the Rhine River suspended particulate matter mass was modeled in response to mean discharge. Natural logarithm transformations were applied to both the explanatory and the response variables. To account for the outliers introduced by the two high discharge events in July and August 2021, a binary dummy variable “flood” was added to each model. Model fits were evaluated using the functions provided by the DHARMa package.^47^ Potential microplastic export patterns and export regime types were assessed as suggested by Musolff et al.^48^ The exponent of the power law relationship (*b*) can indicate dilution of the concentration with increasing discharge (*b* < 0), enrichment (*b* > 0) or highlight a constant export pattern (*b* ∼ 0). To investigate the export regime type, the coefficient of variance (CV) ratio of microplastic number concentration m^–3^ (CV_C_) to discharge (CV_Q_) was used; a ratio of CV_C_/CV_Q_ ≥ 0.5 indicates a chemodynamic regime in which solute load variance is dominated by discharge, while CV_C_/CV_Q_ ≤ 0.5 can be interpreted as a chemostatic regime (i.e., not discharge dominated load variance). Hydrological data were provided by the Federal Office for the Environment (FOEN, Swiss Confederation) and downloaded from the data repository of Basel Stadt (Open BS, data.bs.ch).

To improve comparability of microplastic concentrations with published literature, respective concentrations were aligned to a default microplastic particle size range of 1–5000 μm, as well as 300–5000 μm, employing correction factors calculated according to Koelmans et al. (2020) adopting their estimated exponent (α = 1.6).

### Quality Control

For each sample, a procedural blank was processed in parallel to account for the potential sample contamination. During SPM subsampling, an identical glass container was placed next to the sample container and kept open for the same duration. For microplastic extraction, the control container was rinsed with 10 mL of ultrapure water, and the latter was processed as described above; all reported microplastic concentrations were thus blank corrected by considering polymer types and size classes. Whenever possible, microplastic extraction steps were performed in a clean bench (SKAN AG, Switzerland, model HFX.180BS), and sample vials were kept covered with aluminum foil. Equipment used for sampling and extraction was almost exclusively either glass, metal or wood, and thoroughly rinsed before use with ultrapure water using a PTFE squirt bottle; PTFE cannot be detected in the spectral IR range between 4000–1300 cm^–1^ as used here. The one exception was during the flotation step, where medical grade plastic syringes were used (PP; Codan Medical ApS, Denmark); these were new syringes, and they were rinsed with ultrapure water before use. To further minimize contamination potential from airborne particles, laboratories where microplastic extraction and FTIR analyses took place were equipped with dustboxes (DB1000, G4 prefiltration, HEPA-H14 final filtration, *Q*= 950 m^3^ h^–1^; Möcklinghoff Lufttechnik, Gelsenkirchen, Germany). Wearing of gloves was avoided, except during Fenton’s digestion step, and cotton clothing was worn exclusively.

It is further recommended that a minimum of 500 L be sampled in studies assessing microplastic pollution in environmental studies.^16,49^ For all but two subsamples (91%), analyzed SPM corresponded to a volume exceeding this threshold. The two exceptions were from the high discharge events in July and August 2021, during which sampling time was shortened to prevent overlading of the collection sheet due to increased SPM concentration (Table S1).

## Results

**Figure 2:**
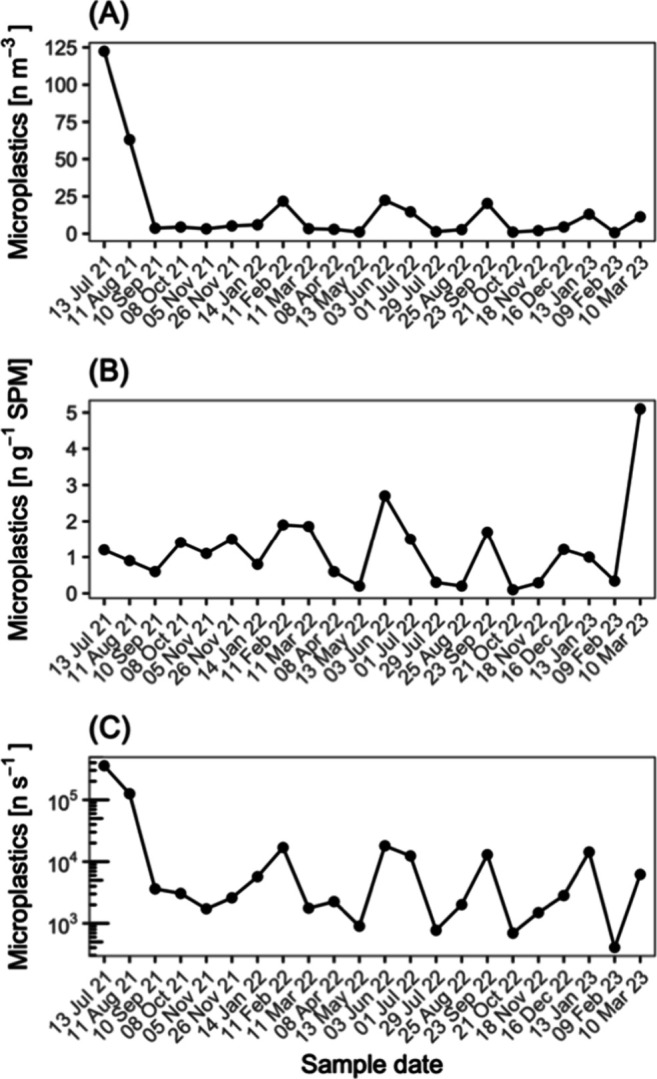
Microplastic in the Rhine River water column near Basel. Concentration in water (A), suspended particulate matter (SPM; B), and estimated load (C).

**Figure 3:**
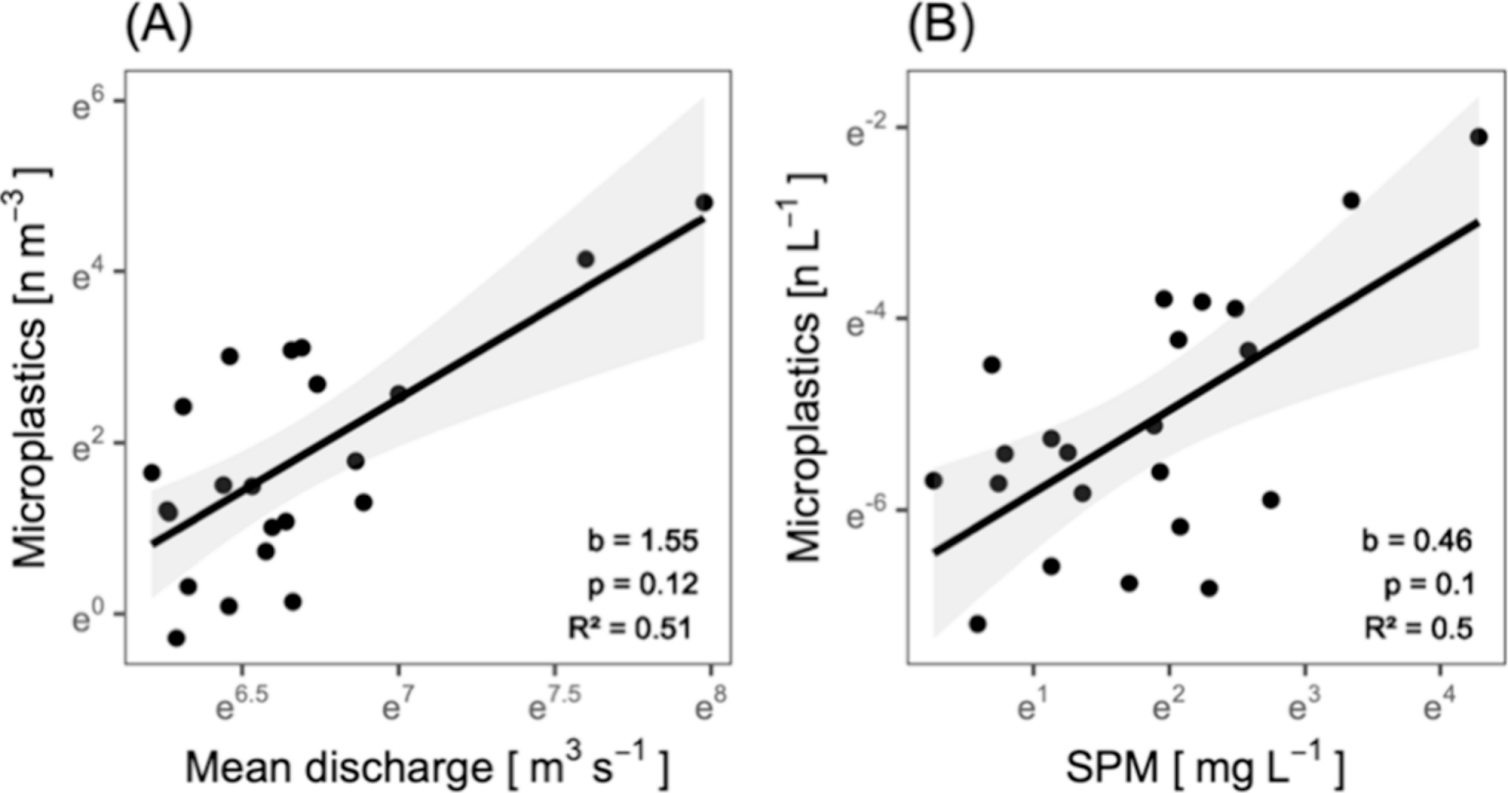
Rhine River microplastic concentration in response to (A) mean discharge during each sampling instance and (B) suspended particulate matter concentration. Slope (b), corresponding *p*-value, and R^2^ are indicated. Hydrological data: Federal Office for the Environment (FOEN).

The samples yielded 219 confirmed microplastic particles. Between July 2021 and March 2023, the number concentration of microplastic particles in the water column varied by 2 orders of magnitude, ranging from 0.75 to 1.23 × 10^2^*n* m^–3^ (median = 4.48 *n* m^–3^; Figure 2A). The estimated mass concentrations ranged from 0 to 3.81 × 10^–4^ g m^–3^ (median = 5.44 × 10^–6^ g m^–3^). Microplastic number concentration in suspended particulate matter was less variable, ranging from 0.1 to 5.1 *n* g^–1^ (median = 1.05 *n* g^–1^; Figure 2B), while mean mass concentration ranged from 0 to 7.89 × 10^–6^ g_plastic_ g^–1^ (median = 7.38 × 10^–7^ g_plastic_ g^–1^). The study period included seasonal variation in discharge and also captured a high discharge period in summer 2021 (Figure S1a); median river discharge during sampling events was 725 m^3^ s^–1^, ranging between 497 m^3^ s^–1^ and 2916 m^3^ s^–1^. The estimated microplastic particle number load was therefore highly variable, ranging between 4.04 × 10^2^*n* s^–1^ and 3.57 × 10^5^*n* s^–1^ (median = 2.94 × 10^3^*n* s^–1^; Figure 2C). Microplastic number concentrations in water were neither well explained by Rhine River discharge in the Basel area (Figure 3A; Table S3), nor the concentration of suspended particulate matter (Figure 3B; Table S4). Note, however, that mean river discharge was identified as a significant predictor of suspended particulate matter (Table S5). The CV_C_/CV_Q_ ratio for microplastic number concentrations m^–3^ was 2.87.

**Figure 4:**
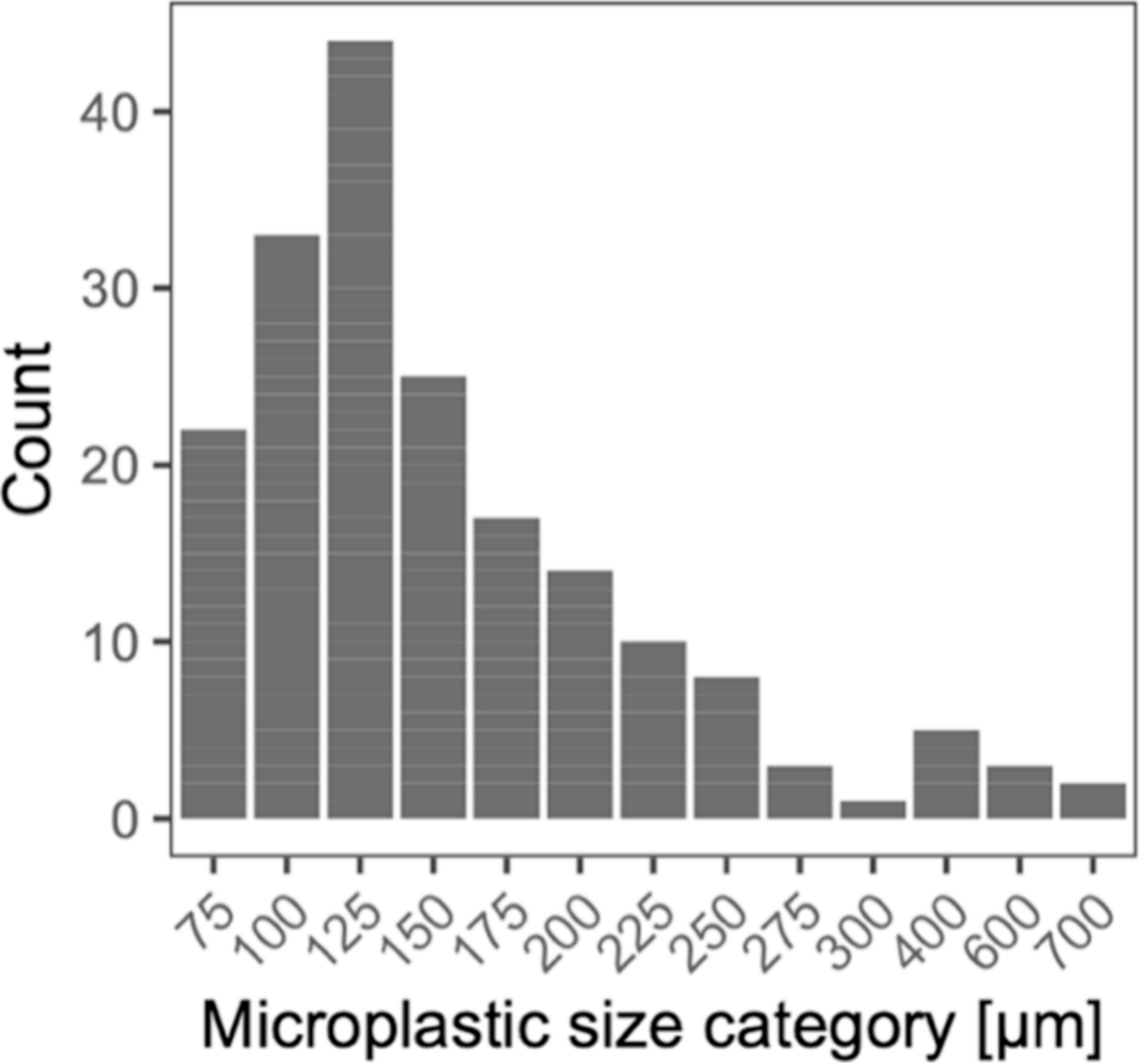
Microplastic particle size distribution for all items identified over the study period.

**Figure 5:**
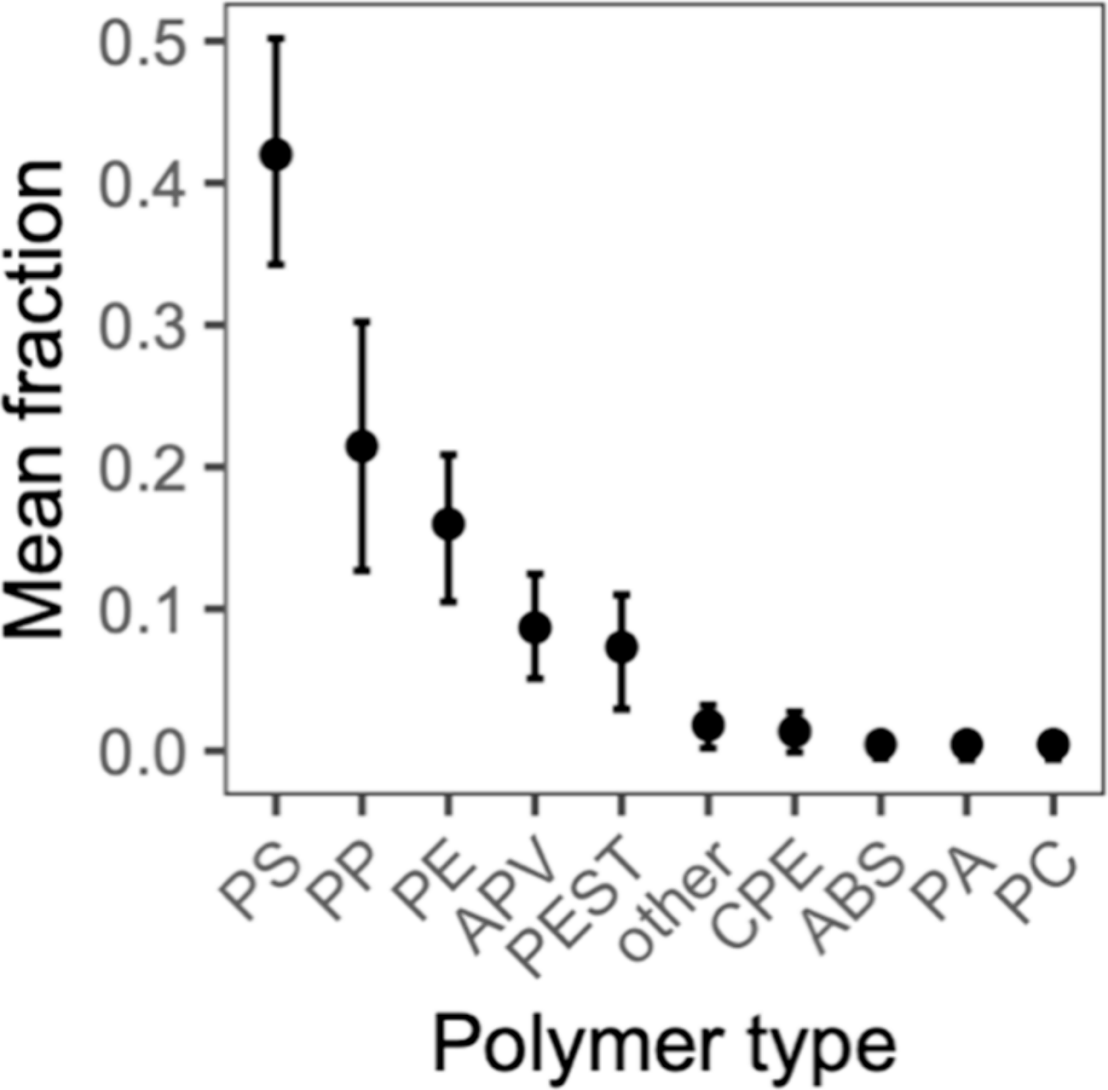
Weighted mean fraction (±95% confidence interval) of microplastic polymer types in suspended matter (SM) samples (*n* = 22) of the water column of the river Rhine. ABS: acrylonitrile butadiene styrene; APV: acrylates, polyurethanes, varnish; CPE: chlorinated polyethylene; PA: polyamide; PC: polycarbonate; PE: polyethylene; PEST: polyester; PP: polypropylene; PS: polystyrene; other: silicone/PDMS, ethylene acrylic acid, styrene butadiene, poly(vinyl stearate).

**Figure 6:**
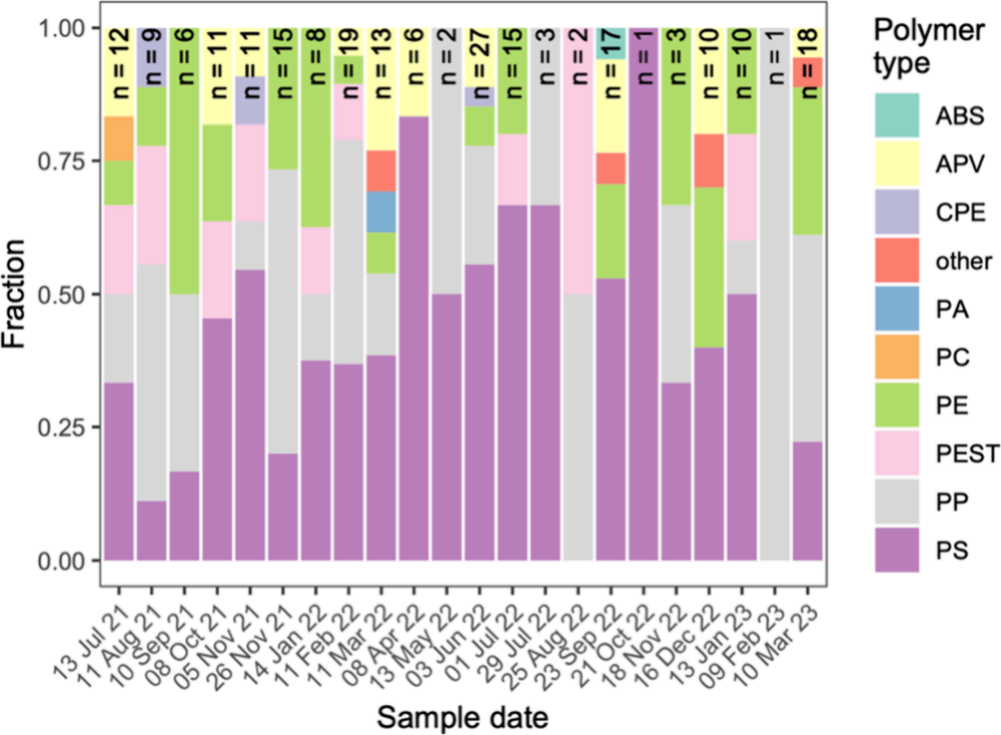
Fraction of polymer types found in Rhine River water column samples from the Basel region. ABS: acrylonitrile butadiene styrene; APV: acrylates, polyurethanes, varnish; CPE: chlorinated polyethylene; PA: polyamide; PC: polycarbonate; PE: polyethylene; PEST: polyester; PP: polypropylene; PS: polystyrene; other: silicone/PDMS, ethylene acrylic acid, styrene butadiene, poly(vinyl stearate). n: total number of particles identified.

Among the isolated particles confirmed as synthetic polymers, nine main polymer types were found. The most common were polystyrene (PS, 42%), polypropylene (PP, 21%), polyethylene (PE, 16%), the group composed of acrylates, polyurethanes and varnishes (APV, 9%), and polyesters (PEST, 7%; Figure 5). Polymer type fractions were variable over time without displaying any trends (Figure 6); for instance, over the study period, the percentage of PS ranged between 0% and 100%. Microplastic particles occurred in a size range between from 50 to 700 μm, with the highest abundance in the 100–125 μm size class (Figure 4); 93.6% of microplastic particles were ≤300 μm. Peak percent abundance was also observed in the particle size distribution of the suspended particulate matter considering particles >50 μm, i.e., the minimum size adopted in the present study (Figure S2).

Using the estimated conversion factor of 11.4 to align the concentrations reported here (50–3000 μm) to a default size range of 1–5000 μm yielded number concentrations ranging between 8.32–1358 *n* m^–3^ (median = 49.7 *n* m^–3^).

## Discussion

This study provides time-resolved data on the pollution of the Rhine River water column in the Basel area by microplastics. We found microplastics in samples from all 22 months, at a median number concentration of 4.48 *n* m^–3^ in water, and 1.05 *n* g^–1^ in suspended particulate matter. Microplastic number concentration in water (*C*) showed a significant positive correlation with the average river discharge (*Q*), if data from a high discharge event in 2021 were included (up to 4× above the median of the study period). Median mass concentration was estimated as 5.44 × 10^–6^ g m^–3^; note, however, that mass estimations based on FTIR data are not as reliable as measured with other techniques.^50^

Comparing concentrations between studies remains challenging due to missing methodological standardization, and targeting of different size ranges,^16,26,32^ but comparability can be improved by aligning reported concentrations to a default size range (1–5000 μm).^45^ The aligned number concentrations found in the present study range from 8.53 *n* m^–3^ to 1393 *n* m^–3^ (median = 50.9 *n* m^–3^). These are lower than what was measured in the water column downstream in The Netherlands, also using a continuous centrifugation system (aligned 405–2027 *n* m^–3^;^21^). The finding is consistent with data from surface water measurements, where concentrations in the impounded Rhine are lower than in sections toward the Rhine delta.^18^ The median number concentration measured in the water column was in a similar range to concentrations previously found in surface water within the area (aligned range: 41.1–123.3 *n* m^–3^;^22^). The dominance of small microplastics, i.e., 93.6% were ≤300 μm, is also congruent with observations from the Netherlands.^21^ If in turn we considered microplastics >300 μm only (*n* = 10), then the concentration extended to a range of 300–5000 μm over the entire study period would have been 0.31 *n* m^–3^, thus 3.55–10.7× lower than the surface water concentration previously measured.^22^ This suggests a decreased incidence of larger particles in the water column in the Basel area, although it is important to note that the measurements stem from different sampling campaigns. In a study where microplastic concentration was assessed using multiple depth sampling,^29^ estimates highlighted that indeed the large (1–4.79 mm) fraction of microplastics in the water column would have been overestimated by up to 95% if extrapolated from surface water samples. To date, however, data on microplastic concentrations in river water column are scarce, and local conditions may exert a strong influence on the vertical distribution of microplastics, as observed in the Milwaukee River basin.^29^

The high discharge event at the start of this study displayed 4.5–11.6× higher concentrations of suspended particulate matter than the observed mean in the ensuing months (6.26 mg L^–1^; Figure S1). Such concentration (*C*)–discharge (*Q*) relationships as identified here have commonly been reported from rivers, and can serve as indicators of catchment-scale processes, e.g., dilution, or enrichment.^48,51^ Our data show that microplastic number concentration in suspended particulate matter varied less, compared to concentration in water (Figure 2), similar to findings of Wagner et al.^52^ Nonetheless, in contrast to the general *C*–*Q* relationship for suspended particulate matter, discharge did not emerge as a statistically significant predictor of microplastic number concentrations in water (Figure 3A). Based on a CV_C_/CV_Q_ ratio of 2.87, we deduce a trend toward a chemodynamic export regime, i.e., load variance not strongly determined by discharge, with a discharge dependent enrichment regime (*b* = 1.55;^48^). Our results thus reflect previous observations of strongly elevated flow conditions resuspending or mobilizing microplastics from river shore and bed sediments,^53−56^ as well as enrichment via diffuse inflows from urban runoff and stormwater after heightened rainfall events.^17^ Our findings based on the water column support previous observations drawn from data on surface water microplastic pollution based on which a clear *C*–*Q* relationship was absent (0.3–5 mm;^22^). The high temporal variability of chemodynamic regimes can perturb river biota, but also promote adaptation with fast recovery.^57−59^ Moreover, it is important to emphasize that even the highest concentration measured here (aligned: 1.36 × 10^3^*n* m^–3^) is about 3 orders of magnitude below estimated environmental effect threshold concentrations at which 10% of species would be affected.^45^ While in some other urban settings microplastic *C*–*Q* relationships have been found,^52,60,61^ there does not appear to be a default microplastic export regime, as multiseason assessments yield inconsistent results that likely reflect the interplay of local factors.^52,62^

The polymer types detected in the suspended particulate matter are frequently identified in environmental water samples, such as PS (42%), PP (21%), and PE (16%; Figure 5;^16,63^). Together, these polymers represent 55.4% of the demand among plastic converters, and are predominantly used for products with short life cycles, such as packaging,^64^ which leads to these being overrepresented among discarded polymers.^1^ In the water column, a vertical polymer type distribution may be expected based on polymer properties, with particles less dense than water residing in surface water, and denser particles settling out of the water column over time. While this has been observed in a marine setting,^30^ several additional factors interact to influence vertical microplastic particle distribution, such as biofouling,^65,66^ particle aggregation,^26,67,68^ and turbulent mixing.^31,53^ In a study designed to assess microplastic distribution in river water column, Lenaker et al.^29^ indeed reported particle segregation by polymer type. Interestingly, the most prevalent microplastic polymer identified in the present study was PS, while a previous assessment of surface water in the Basel area had shown PE to be most abundant among small microplastics (0.3–1 mm), while the large fraction (1–5 mm) had been dominated by PS (ca. 50%).^22^ Although PS is denser than water (ρ = 1.05 g cm^–3^), it had primarily been found as a foam. Compared with PE and PP, foamed PS exhibits significantly higher fragmentation rates,^69^ which may explain the dominance of PS in the water column observed here, constituting fragmented foamed PS. Nonetheless, high prevalence of specific polymer types in Rivers also emerge as consequence of local input sources, and have been observed to vary substantially at a greater spatial resolution.^26^ Moreover, we observed a higher incidence of particles from the polyurethanes and varnishes group than previous studies of surface waters,^18,22^ which likely constitute ship paint.^70^ Note, however, that surface water sampling with Manta nets may severely underestimate the concentration of such paint particles.^71^ Ship paint particles had been expected due to the commercial shipping harbor situated upstream of the sampling site. The prevalence found here (9%) was significantly lower than in benthic midstream sediments from the Rhine downstream (70%;^20^), and we therefore interpret our findings to represent an intermediate polymer type mix between observations from surface waters and benthic sediments.

The present study, characterized by its opportunistic nature, acknowledges certain limitations, particularly in relation to the employed sampling methodology. Flow through centrifugation, a well-established method for collecting suspended particulate matter, still introduces uncertainties regarding the efficiency of extracting negatively buoyant polymer types. A pilot study conducted elsewhere using PET, PP, PE, PVDC and expanded PS, demonstrated recovery rates of at least 95%.^72^ However, it is noteworthy that although centrifugation was conducted at 17,000 rpm, the G-forces differed (approximately 24,000 G in Hildebrandt et al. vs 21000 G used here), potentially influencing settling conditions. Furthermore, factors such as biofilm formation and aggregation with other organic and inorganic particles contribute to the settling behavior of microplastics in aquatic environments, which may affect behavior during centrifugation—an aspect that merits further investigation. While Hildebrandt et al.^72^ did not notice any effects of centrifugation on particle size distributions, it may be worth investigating whether this holds true for water samples containing inorganic particles, such as silt, in future studies.

Microplastic concentrations in river water can be highly variable. The present study meets a research gap in the context of time-resolved microplastic pollution in one of Europe’s main rivers, and provides, to date, rare data on water column microplastic loads. We show that median microplastic number concentrations in the water column are comparable to concentrations measured in surface waters previously. Also, in accordance with previous studies from the impounded Rhine River section, microplastic number concentration in water is not well explained by river discharge, suggesting a trend toward a chemodynamic export regime. Occasionally, however, high discharge events can cause temporary spikes in microplastic loads, as reported herein. Nonetheless, measured peak concentrations remained 3 orders of magnitude below estimated effect concentrations for >90% of biota.^45^

## Supporting Information Available

Two figures (Rhine River discharge and suspended particulate matter by dry weight over the study period; Mean (*n* = 9) particle size distribution of untreated suspended particulate matter for selected samples), and five tables (sampling meta data, three statistical summary tables, and synthetic polymer densities used for mass estimation) (PDF)
Scripts used for the study (ZIP); raw data (DOI: 10.17632/k2kvf949ht.1)

## References

[ref1] Geyer R.; Jambeck J. R.; Law K. L. Production, Use, and Fate of All Plastics Ever Made. Sci. Adv. 2017, 3 (7), e1700782 10.1126/sciadv.1700782. 28776036 PMC5517107

[ref2] Brahney J.; Hallerud M.; Heim E.; Hahnenberger M.; Sukumaran S. Plastic Rain in Protected Areas of the United States. Science. 2020, 368 (6496), 1257–1260. 10.1126/science.aaz5819. 32527833

[ref3] Peeken I.; Primpke S.; Beyer B.; Gütermann J.; Katlein C.; Krumpen T.; Bergmann M.; Hehemann L.; Gerdts G. Arctic Sea Ice Is an Important Temporal Sink and Means of Transport for Microplastic. Nat. Commun.. 2018, 9. 10.1038/s41467-018-03825-5. PMC5915590 29692405

[ref4] Lavers J. L.; Bond A. L. Exceptional and Rapid Accumulation of Anthropogenic Debris on One of the World’s Most Remote and Pristine Islands. Proc. Natl. Acad. Sci. U. S. A. 2017, 114 (23), 6052–6055. 10.1073/pnas.1619818114. 28507128 PMC5468685

[ref5] Koelmans A. A.; Redondo-Hasselerharm P. E.; Nor N. H. M.; de Ruijter V. N.; Mintenig S. M.; Kooi M. Risk Assessment of Microplastic Particles. Nat. Rev. Mater. 2022, 7, 138–152. 10.1038/s41578-021-00411-y.

[ref6] Galloway T. S.; Cole M.; Lewis C. Interactions of Microplastic Debris throughout the Marine Ecosystem. Nat. Ecol. Evol. 2017, 1, 0116 10.1038/s41559-017-0116. 28812686

[ref7] de Ruijter V. N.; Redondo-Hasselerharm P. E.; Gouin T.; Koelmans A. A. Quality Criteria for Microplastic Effect Studies in the Context of Risk Assessment: A Critical Review. Environ. Sci. Technol. 2020, 54, 11692–11705. 10.1021/acs.est.0c03057. 32856914 PMC7547869

[ref8] Ogonowski M.; Wagner M.; Rogell B.; Haave M.; Lusher A. Microplastics Could Be Marginally More Hazardous than Natural Suspended Solids - A Meta-Analysis. Ecotoxicol. Environ. Saf. 2023, 264, 115406 10.1016/j.ecoenv.2023.115406. 37639826

[ref9] Koelmans A. A.; Diepens N. J.; Mohamed Nor N. H.; Bank M. S. Weight of Evidence for the Microplastic Vector Effect in the Context of Chemical Risk Assessment. In Microplastic in the Environment: Pattern and Process, Ed.; 2022; pp 155–197. 10.1007/978-3-030-78627-4_6.

[ref10] Waldschläger K.; Brückner M. Z. M.; Carney Almroth B.; Hackney C. R.; Adyel T. M.; Alimi O. S.; Belontz S. L.; Cowger W.; Doyle D.; Gray A.; Kane I.; Kooi M.; Kramer M.; Lechthaler S.; Michie L.; Nordam T.; Pohl F.; Russell C.; Thit A.; Umar W.; Valero D.; Varrani A.; Warrier A. K.; Woodall L. C.; Wu N. Learning from Natural Sediments to Tackle Microplastics Challenges: A Multidisciplinary Perspective. Earth-Science Rev. 2022, 228, 104021 10.1016/j.earscirev.2022.104021.

[ref11] Stubbins A.; Law K. L.; Muñoz S. E.; Bianchi T. S.; Zhu L. Plastics in the Earth System. Science. 2021, 373 (6550), 51–55. 10.1126/science.abb0354. 34210876

[ref12] Meijer L. J. J.; van Emmerik T.; van der Ent R.; Schmidt C.; Lebreton L. More than 1000 Rivers Account for 80% of Global Riverine Plastic Emissions into the Ocean. Sci. Adv. 2021, 7 (18), eaaz5803 10.1126/sciadv.aaz5803. 33931460 PMC8087412

[ref13] Weiss L.; Ludwig W.; Heussner S.; Canals M.; Ghiglione J.-F.; Estournel C.; Constant M.; Kerhervé P. The Missing Ocean Plastic Sink: Gone with the Rivers. Science. 2021, 373, 107–111. 10.1126/science.abe0290. 34210886

[ref14] Kaandorp M. L. A.; Lobelle D.; Kehl C.; Dijkstra H. A.; van Sebille E. Global Mass of Buoyant Marine Plastics Dominated by Large Long-Lived Debris. Nat. Geosci. 2023, 16, 689–694. 10.1038/s41561-023-01216-0.

[ref15] Lebreton L. C. M.; van der Zwet J.; Damsteeg J.-W.; Slat B.; Andrady A.; Reisser J. River Plastic Emissions to the World’s Oceans. Nat. Commun. 2017, 8, 15611 10.1038/ncomms15611. 28589961 PMC5467230

[ref16] Lofty J.; Ouro P.; Wilson C. A. M. E. Microplastics in the Riverine Environment: Meta-Analysis and Quality Criteria for Developing Robust Field Sampling Procedures. Sci. Total Environ. 2023, 863, 160893 10.1016/j.scitotenv.2022.160893. 36516921

[ref17] Wang C.; O’Connor D.; Wang L.; Wu W. M.; Luo J.; Hou D. Microplastics in Urban Runoff: Global Occurrence and Fate. Water Res. 2022, 225, 119129 10.1016/j.watres.2022.119129. 36170770

[ref18] Mani T.; Hauk A.; Walter U.; Burkhardt-Holm P. Microplastics Profile along the Rhine River. Sci. Rep. 2016, 5, 17988 10.1038/srep17988. PMC4672315 26644346

[ref19] Klein S.; Worch E.; Knepper T. P. Occurrence and Spatial Distribution of Microplastics in River Shore Sediments of the Rhine-Main Area in Germany. Environ. Sci. Technol. 2015, 49, 6070–6076. 10.1021/acs.est.5b00492. 25901760

[ref20] Mani T.; Primpke S.; Lorenz C.; Gerdts G.; Burkhardt-Holm P. Microplastic Pollution in Benthic Midstream Sediments of the Rhine River. Environ. Sci. Technol. 2019, 53 (10), 6053–6062. 10.1021/acs.est.9b01363. 31021624

[ref21] Leslie H. A.; Brandsma S. H.; van Velzen M. J. M.; Vethaak A. D. Microplastics En Route: Field Measurements in the Dutch River Delta and Amsterdam Canals, Wastewater Treatment Plants, North Sea Sediments and Biota. Environ. Int. 2017, 101, 133–142. 10.1016/j.envint.2017.01.018. 28143645

[ref22] Mani T.; Burkhardt-Holm P. Seasonal Microplastics Variation in Nival and Pluvial Stretches of the Rhine River - From the Swiss Catchment towards the North Sea. Sci. Total Environ. 2020, 707, 135579 10.1016/j.scitotenv.2019.135579. 31784148

[ref23] Cheung P. K.; Hung P. L.; Fok L. River Microplastic Contamination and Dynamics upon a Rainfall Event in Hong Kong, China. Environ. Process. 2019, 6, 253–264. 10.1007/s40710-018-0345-0.

[ref24] Schmidt L. K.; Bochow M.; Imhof H. K.; Oswald S. E. Multi-Temporal Surveys for Microplastic Particles Enabled by a Novel and Fast Application of SWIR Imaging Spectroscopy - Study of an Urban Watercourse Traversing the City of Berlin, Germany. Environ. Pollut. 2018, 239, 579–589. 10.1016/j.envpol.2018.03.097. 29684884

[ref25] Talbot R.; Chang H. Microplastics in Freshwater: A Global Review of Factors Affecting Spatial and Temporal Variations. Environ. Pollut. 2022, 292, 118393 10.1016/j.envpol.2021.118393. 34678395

[ref26] Skalska K.; Ockelford A.; Ebdon J. E.; Cundy A. B. Riverine Microplastics: Behaviour, Spatio-Temporal Variability, and Recommendations for Standardised Sampling and Monitoring. J. Water Process Eng. 2020, 38, 101600 10.1016/j.jwpe.2020.101600.

[ref27] Harris P. T.; Maes T.; Raubenheimer K.; Walsh J. P. A Marine Plastic Cloud - Global Mass Balance Assessment of Oceanic Plastic Pollution. Cont. Shelf Res. 2023, 255, 104947 10.1016/j.csr.2023.104947.

[ref28] Cowger W.; Gray A. B.; Guilinger J. J.; Fong B.; Waldschläger K. Concentration Depth Profiles of Microplastic Particles in River Flow and Implications for Surface Sampling. Environ. Sci. Technol. 2021, 55 (9), 6032–6041. 10.1021/acs.est.1c01768. 33896174

[ref29] Lenaker P. L.; Baldwin A. K.; Corsi S. R.; Mason S. A.; Reneau P. C.; Scott J. W. Vertical Distribution of Microplastics in the Water Column and Surficial Sediment from the Milwaukee River Basin to Lake Michigan. Environ. Sci. Technol. 2019, 53 (21), 12227–12237. 10.1021/acs.est.9b03850. 31618011

[ref30] Choy C. A.; Robison B. H.; Gagne T. O.; Erwin B.; Firl E.; Halden R. U.; Hamilton J. A.; Katija K.; Lisin S. E.; Rolsky C.; S Van Houtan K. The Vertical Distribution and Biological Transport of Marine Microplastics across the Epipelagic and Mesopelagic Water Column. Sci. Rep. 2019, 9, 7843 10.1038/s41598-019-44117-2. 31171833 PMC6554305

[ref31] Pabortsava K.; Lampitt R. S. High Concentrations of Plastic Hidden beneath the Surface of the Atlantic Ocean. Nat. Commun. 2020, 11 (1), 1–11. 10.1038/s41467-020-17932-9. 32811835 PMC7434887

[ref32] Liu K.; Courtene-Jones W.; Wang X.; Song Z.; Wei N.; Li D. Elucidating the Vertical Transport of Microplastics in the Water Column: A Review of Sampling Methodologies and Distributions. Water Res. 2020, 186, 116403 10.1016/j.watres.2020.116403. 32932095

[ref33] Frings R. M.; Hillebrand G.; Gehres N.; Banhold K.; Schriever S.; Hoffmann T. From Source to Mouth: Basin-Scale Morphodynamics of the Rhine River. Earth-Science Rev. 2019, 196, 102830 10.1016/j.earscirev.2019.04.002.

[ref34] IKSR. Internationale Flussgebietseinheit Rhein - Merkmale, Überprüfung Der Umweltauswirkungen Menschlicher Tätigkeiten Und Wirtschaftliche Analyse Der Wassernutzung; 2005.

[ref35] Abel S. M.; Primpke S.; Wu F.; Brandt A.; Gerdts G. Human Footprints at Hadal Depths: Interlayer and Intralayer Comparison of Sediment Cores from the Kuril Kamchatka Trench. Sci. Total Environ. 2022, 838, 156035 10.1016/j.scitotenv.2022.156035. 35598673

[ref36] Hurley R. R.; Lusher A. L.; Olsen M.; Nizzetto L. Validation of a Method for Extracting Microplastics from Complex, Organic-Rich, Environmental Matrices. Environ. Sci. Technol. 2018, 52 (13), 7409–7417. 10.1021/acs.est.8b01517. 29886731

[ref37] Scheurer M.; Bigalke M. Microplastics in Swiss Floodplain Soils. Environ. Sci. Technol. 2018, 52 (6), 3591–3598. 10.1021/acs.est.7b06003. 29446629

[ref38] Cowger W.; Steinmetz Z.; Gray A.; Munno K.; Lynch J.; Hapich H.; Primpke S.; De Frond H.; Rochman C.; Herodotou O. Microplastic Spectral Classification Needs an Open Source Community: Open Specy to the Rescue!. Anal. Chem. 2021, 93 (21), 7543–7548. 10.1021/acs.analchem.1c00123. 34009953

[ref39] Chabuka B. K.; Kalivas J. H. Application of a Hybrid Fusion Classification Process for Identification of Microplastics Based on Fourier Transform Infrared Spectroscopy. Appl. Spectrosc. 2020, 74 (9), 1167–1183. 10.1177/0003702820923993. 32297518

[ref40] Primpke S.; Wirth M.; Lorenz C.; Gerdts G. Reference Database Design for the Automated Analysis of Microplastic Samples Based on Fourier Transform Infrared (FTIR) Spectroscopy. Anal. Bioanal. Chem. 2018, 410 (21), 5131–5141. 10.1007/s00216-018-1156-x. 29978249 PMC6113679

[ref41] Smith B. C. Fundamentals of Fourier Transform Infrared Spectroscopy; CRC Press LLC: Boca Raton, 2011.

[ref42] Cowger W.; Gray A.; Christiansen S. H.; DeFrond H.; Deshpande A. D.; Hemabessiere L.; Lee E.; Mill L.; Munno K.; Ossmann B. E.; Pittroff M.; Rochman C.; Sarau G.; Tarby S.; Primpke S. Critical Review of Processing and Classification Techniques for Images and Spectra in Microplastic Research. Appl. Spectrosc. 2020, 74 (9), 989–1010. 10.1177/0003702820929064. 32500727

[ref43] Primpke S.; Lorenz C.; Rascher-Friesenhausen R.; Gerdts G. An Automated Approach for Microplastics Analysis Using Focal Plane Array (FPA) FTIR Microscopy and Image Analysis. Anal. Methods 2017, 9 (9), 1499–1511. 10.1039/C6AY02476A.

[ref44] Simon M.; van Alst N.; Vollertsen J. Quantification of Microplastic Mass and Removal Rates at Wastewater Treatment Plants Applying Focal Plane Array (FPA)-Based Fourier Transform Infrared (FT-IR) Imaging. Water Res. 2018, 142, 1–9. 10.1016/j.watres.2018.05.019. 29804032

[ref45] Koelmans A. A.; Redondo-Hasselerharm P. E.; Mohamed Nor N. H.; Kooi M. Solving the Nonalignment of Methods and Approaches Used in Microplastic Research to Consistently Characterize Risk. Environ. Sci. Technol. 2020, 54 (19), 12307–12315. 10.1021/acs.est.0c02982. 32885967 PMC7547870

[ref46] Canty A.; Ripley B. Boot: Bootstrap R (S-Plus) Functions. 2022.

[ref47] Hartig F. DHARMa: Residual Diagnostics for Hierarchical (Multi-Level/Mixed) Regression Models. R Package Version 0.4.3. 2021.

[ref48] Musolff A.; Fleckenstein J. H.; Rao P. S. C.; Jawitz J. W. Emergent Archetype Patterns of Coupled Hydrologic and Biogeochemical Responses in Catchments. Geophys. Res. Lett. 2017, 44 (9), 4143–4151. 10.1002/2017GL072630.

[ref49] Koelmans A. A.; Mohamed Nor N. H.; Hermsen E.; Kooi M.; Mintenig S. M.; De France J. Microplastics in Freshwaters and Drinking Water: Critical Review and Assessment of Data Quality. Water Res. 2019, 155, 410–422. 10.1016/j.watres.2019.02.054. 30861380 PMC6449537

[ref50] Primpke S.; Fischer M.; Lorenz C.; Gerdts G.; Scholz-Böttcher B. M. Comparison of Pyrolysis Gas Chromatography/Mass Spectrometry and Hyperspectral FTIR Imaging Spectroscopy for the Analysis of Microplastics. Anal. Bioanal. Chem. 2020, 412 (30), 8283–8298. 10.1007/s00216-020-02979-w. 33104827 PMC7680748

[ref51] Müller G.; Förstner U. General Relationship between Suspended Sediment Concentration and Water Discharge in the Alpenrhein and Some Other Rivers. Nature 1968, 217, 244–245. 10.1038/217244a0.

[ref52] Wagner S.; Klöckner P.; Stier B.; Römer M.; Seiwert B.; Reemtsma T.; Schmidt C. Relationship between Discharge and River Plastic Concentrations in a Rural and an Urban Catchment. Environ. Sci. Technol. 2019, 53 (17), 10082–10091. 10.1021/acs.est.9b03048. 31380631

[ref53] Haberstroh C. J.; Arias M. E.; Yin Z.; Wang M. C. Effects of Hydrodynamics on the Cross-Sectional Distribution and Transport of Plastic in an Urban Coastal River. Water Environ. Res. 2021, 93 (2), 186–200. 10.1002/wer.1386. 32609913

[ref54] Nel H. A.; Dalu T.; Wasserman R. J. Sinks and Sources: Assessing Microplastic Abundance in River Sediment and Deposit Feeders in an Austral Temperate Urban River System. Sci. Total Environ. 2018, 612, 950–956. 10.1016/j.scitotenv.2017.08.298. 28886547

[ref55] Eerkes-Medrano D.; Thompson R.; Zeng E. Y. Occurrence, Fate, and Effect of Microplastics in Freshwater Systems. Microplastic Contamination in Aquatic Environments: An Emerging Matter of Environmental Urgency 2018, 95–132. 10.1016/B978-0-12-813747-5.00004-7.

[ref56] Hurley R.; Woodward J.; Rothwell J. J. Microplastic Contamination of River Beds Significantly Reduced by Catchment-Wide Flooding. Nat. Geosci. 2018, 11, 251–257. 10.1038/s41561-018-0080-1.

[ref57] Giger W. The Rhine Red, the Fish Dead—the 1986 Schweizerhalle Disaster, a Retrospect and Long-Term Impact Assessment. Environ. Sci. Pollut. Res. 2009, 16, 98–111. 10.1007/s11356-009-0156-y. 19479296

[ref58] Lake P. S. Disturbance, Patchiness, and Diversity in Streams. J. North Am. Benthol. Soc. 2000, 19 (4), 573–592. 10.2307/1468118.

[ref59] Naiman R. J.; Decamps H.; Pollock M. The Role of Riparian Corridors in Maintaining Regional Biodiversity. Ecol. Appl. 1993, 3 (2), 209–212. 10.2307/1941822. 27759328

[ref60] Baldwin A. K.; Corsi S. R.; Mason S. A. Plastic Debris in 29 Great Lakes Tributaries: Relations to Watershed Attributes and Hydrology. Environ. Sci. Technol. 2016, 50, 10377–10385. 10.1021/acs.est.6b02917. 27627676

[ref61] Chen H. L.; Gibbins C. N.; Selvam S. B.; Ting K. N. Spatio-Temporal Variation of Microplastic along a Rural to Urban Transition in a Tropical River. Environ. Pollut. 2021, 289, 117895 10.1016/j.envpol.2021.117895. 34364115

[ref62] van Emmerik T.; Strady E.; Kieu-Le T. C.; Nguyen L.; Gratiot N. Seasonality of Riverine Macroplastic Transport. Sci. Rep. 2019, 9, 13549 10.1038/s41598-019-50096-1. 31537881 PMC6753078

[ref63] Erni-Cassola G.; Zadjelovic V.; Gibson M. I.; Christie-Oleza J. A. Distribution of Plastic Polymer Types in the Marine Environment; A Meta-Analysis. J. Hazard. Mater. 2019, 369, 691–698. 10.1016/j.jhazmat.2019.02.067. 30826562

[ref64] PlasticsEurope. Plastics - the Facts 2022. PlasticsEurope 2022.

[ref65] Wright R. J.; Erni-Cassola G.; Zadjelovic V.; Latva M.; Christie-Oleza J. A. Marine Plastic Debris: A New Surface for Microbial Colonization. Environ. Sci. Technol. 2020, 54 (19), 11657–11672. 10.1021/acs.est.0c02305. 32886491

[ref66] Miao L.; Gao Y.; Adyel T. M.; Huo Z.; Liu Z.; Wu J.; Hou J. Effects of Biofilm Colonization on the Sinking of Microplastics in Three Freshwater Environments. J. Hazard. Mater. 2021, 413, 125370 10.1016/j.jhazmat.2021.125370. 33609862

[ref67] Filella M. Questions of Size and Numbers in Environmental Research on Microplastics: Methodological and Conceptual Aspects. Environ. Chem. 2015, 12 (5), 527–538. 10.1071/EN15012.

[ref68] Porter A.; Lyons B. P.; Galloway T. S.; Lewis C. N. The Role of Marine Snows in Microplastic Fate and Bioavailability. Environ. Sci. Technol. 2018, 52, 7111–7119. 10.1021/acs.est.8b01000. 29782157

[ref69] Song Y. K.; Hong S. H.; Jang M.; Han G. M.; Jung S. W.; Shim W. J. Combined Effects of UV Exposure Duration and Mechanical Abrasion on Microplastic Fragmentation by Polymer Type. Environ. Sci. Technol. 2017, 51 (8), 4368–4376. 10.1021/acs.est.6b06155. 28249388

[ref70] Leistenschneider C.; Burkhardt-Holm P.; Mani T.; Primpke S.; Taubner H.; Gerdts G. Microplastics in the Weddell Sea (Antarctica): A Forensic Approach for Discrimination between Environmental and Vessel-Induced Microplastics. Environ. Sci. Technol. 2021, 55, 15900–15911. 10.1021/acs.est.1c05207. 34841863

[ref71] Song Y. K.; Hong S. H.; Jang M.; Kang J. H.; Kwon O. Y.; Han G. M.; Shim W. J. Large Accumulation of Micro-Sized Synthetic Polymer Particles in the Sea Surface Microlayer. Environ. Sci. Technol. 2014, 48 (16), 9014–9021. 10.1021/es501757s. 25059595

[ref72] Hildebrandt L.; Voigt N.; Zimmermann T.; Reese A.; Proefrock D. Evaluation of Continuous Flow Centrifugation as an Alternative Technique to Sample Microplastic from Water Bodies. Mar. Environ. Res. 2019, 151, 104768 10.1016/j.marenvres.2019.104768. 31519451

[ref73] Rheinüberwachungsstation Weil am Rhein (RÜS). https://www.aue.bs.ch/umweltanalytik/rheinueberwachungsstation-weil-am-rhein.html.

