# Supplementary material for: Microplastics in the Water Column of the Rhine River Near Basel: 22 Months of Sampling
Source: Environ Sci Technol. 2024 Mar 13;58(12):5491–9. doi: 10.1021/acs.est.3c08364 (PMC10976896; doi:10.1021/acs.est.3c08364)
Supplement: Supplementary file 1 — es3c08364_si_001.pdf [file es3c08364_si_001.pdf]

SUPPLEMENTARY INFORMATION

Microplastics in the water column of the Rhine River near Basel: 22 months of sampling

Gabriel Erni-Cassola[1*], Reto Dolf[2], Patricia Burkhardt-Holm[1*]

[1]Man-Society-Environment (Programme MGU), Department of Environmental Sciences, University of Basel, Vesalgasse 1, CH-4051 Basel, Switzerland

[2]Abteilung Umweltlabor, Amt für Umwelt und Energie, Department für Wirtschaft, Soziales und Umwelt des Kantons Basel-Stadt, Spiegelgasse 15, Postfach, CH-4001 Basel, Switzerland

[*]Corresponding authors: gabriel.ernicassola@unibas.ch, patricia.holm@unibas.ch

Figures and Tables

Figure S1. Rhine River discharge and suspended particulate matter by dry weight over the study period.
Figure S2. Mean (n = 9) particle size distribution of untreated suspended particulate matter for selected samples.

Table S1. Sampling data, with centrifuge runtime, total sampled volume, suspended particulate matter (SPM) wet weight (WW), and dry weight (DW).
Table S2. Identified polymer types and corresponding density used to estimate particle mass.
Table S3. Statistical summary for estimated microplastic concentration in Rhine River water in response to mean discharge.
Table S4. Statistical summary for estimated microplastic concentration in Rhine River water in response to suspended matter mass concentration in water.
Table S5. Statistical summary for suspended matter mass concentration in water in response to mean discharge.

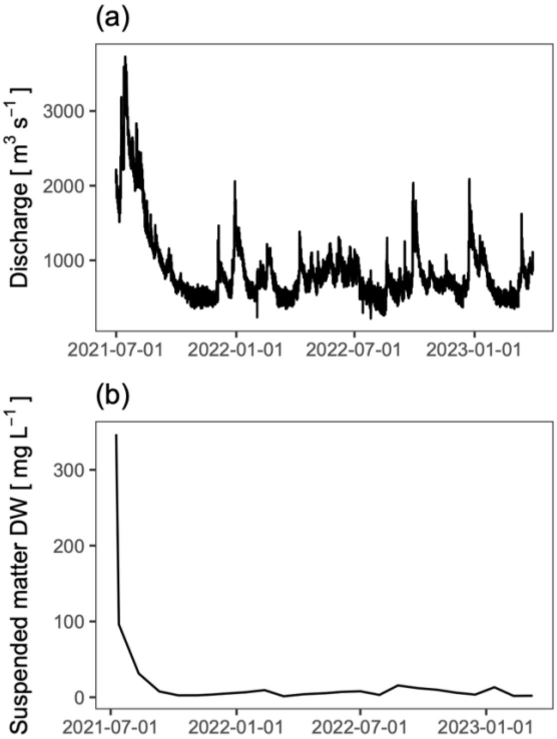

Figure S1. Rhine River discharge (a) and suspended matter by dry weight (DW; b) during study period. Hydrological data: Federal Office for the Environment (FOEN).

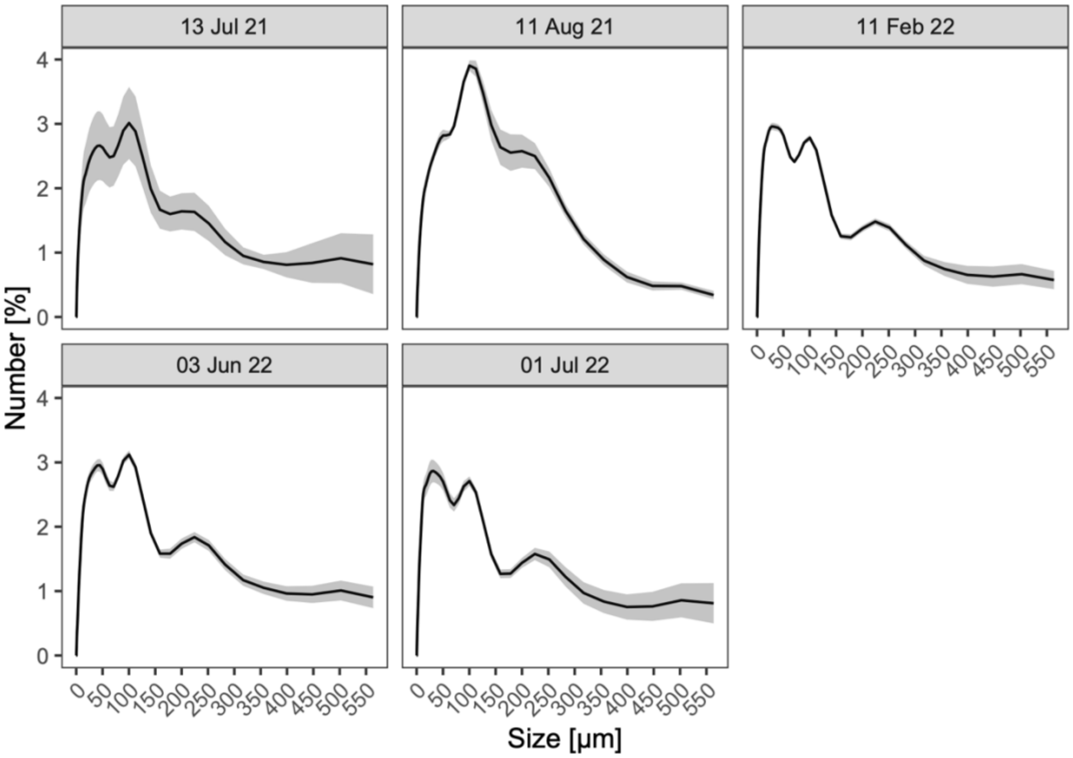

Figure S2. Mean (n = 9) particle size distribution of untreated suspended particulate matter for selected samples. Shading represents standard deviation. Note that July and August 2021 samples stem from high discharge events.

Table S1. Sampling data, with centrifuge runtime, total sampled volume, suspended particulate matter (SPM) wet weight (WW), and dry weight (DW).

Date	Runtime [h]	Total V [m^3]	Total WW SPM [g]	WW SPM Subsample [g]	DW SPM Subsample [g]	DW SPM Analysed [g]	V Corresponding to Analysed SPM [m^3]	DW SPM [mg L^{-1}]
13 Jul 21	12.0	2.2	347	85	55.6	10.0	0.10*	102
11 Aug 21	18.5	3.8	405	49	32	10.0	0.14*	70.1
10 Sep 21	96.0	19.6	243	26	12.9	10.0	1.63	6.15
08 Oct 21	95.0	18.8	108	16.4	7.8	7.8	2.84	2.74
05 Nov 21	92.0	21.5	141	28.7	13	10.0	3.36	2.97
26 Nov 21	96.0	22.5	166	24	11.3	10.0	2.88	3.48
14 Jan 22	93.0	20.1	254	29.6	15.6	10.0	1.50	6.67
11 Feb 22	89.0	19.2	349	59.6	32.1	10.0	1.02	9.79
11 Mar 22	98.0	21.8	78.9	17	7	7.0	4.69	1.49
08 Apr 22	88.5	20.2	187	26.6	12.3	10.0	2.33	4.30
13 May 22	98.0	21.2	236	38	16.9	10.0	2.01	4.96
03 Jun 22	92.5	18.3	249	43	22.4	10.0	1.42	7.06
01 Jul 22	88.0	19.5	280	49.1	28.4	10.0	1.20	8.30
29 Jul 22	97.5	19.9	139	31	16.5	9.9	2.66	3.72
25 Aug 22	71.5	16.1	452	24.1	11.1	9.9	0.76	12.9
23 Sep 22	74.0	16.4	353	35	17.7	10.0	0.92	10.8
21 Oct 22	65.0	16.8	317	47.8	24.1	10.0	1.05	9.52
18 Nov 22	96.0	20.7	269	40.3	19.3	10.3	1.66	6.20
16 Dec 22	70.0	16.4	147	23	8.25	8.3	2.56	3.23
13 Jan 23	91.5	22.0	527	65	35.4	10.0	0.77	13.1
09 Feb 23	64.0	14.6	95.6	8.7	2.94	2.9	1.33	2.21
10 Mar 23	96.0	21.9	137	10	3.53	3.5	1.60	2.21

*Volume below recommended minimum of 0.5 m^3 due to high SPM concentration preventing longer centrifuge runtime.

Table S2. Identified polymer types and corresponding density used to estimate particle mass.

**Polymer**	**Abbreviation**	**Density [g cm^{-3}]**
Polypropylene	PP	0.905[1]
Polyethylene	PE	0.935[1]
Varnish	–	0.94[2]
Polystyrene	PS	1.048[1]
Acrylonitrile butadiene styrene	ABS	1.052[2]
Polyurethane	PU	1.087[1]
Polycarbonate	PC	1.2[1]
Acrylate	–	1.22[1]
Polyamide	PA	1.27[1]
Polyester	PEST	1.38[1]
Chlorinated PE	CPE	1.63[2]

[1](Simon et al., 2018)
[2](Primpke et al., 2020)

Table S3. Estimated microplastic concentration, 95% confidence intervals (CI) and levels of significance (*p*) for a linear model of microplastic concentration in Rhine River water in response to mean discharge during each sampling event and flood.

Predictors	*Estimates*	*CI*	*p*
(Intercept)	-8.63	-21.63 – 4.37	0.181
ln(Mean discharge)	1.55	-0.44 – 3.54	0.119
Flood*	1.04	-1.86 – 3.94	0.463
Observations	22		
R^2/ R^2 adjusted	0.506 / 0.454		

* Dummy variable added to account for outliers during two high discharge events in July and August 2021.

Table S4. Estimated microplastic concentration, 95% confidence intervals (CI) and levels of significance (*p*) for a linear model of microplastic concentration in Rhine River water in response to suspended matter (SM) concentration during each sampling event and flood.

Predictors	*Estimates*	*CI*	*p*
(Intercept)	-6.14	-7.24 – -5.04	**<0.001**
ln(SM concentration)	0.46	-0.16 – 1.09	0.137
Flood*	1.94	-0.12 – 4.00	0.064
Observations	22		
R^2 / R^2 adjusted	0.500 / 0.447		

* Dummy variable added to account for outliers during two high discharge events in July and August 2021.

Table S5. Estimated Rhine River suspended matter concentration, 95% confidence intervals (CI) and levels of significance (*p*) for a linear model using as predictors mean discharge during each sampling event and flood.

Predictors	*Estimates*	*CI*	*p*
(Intercept)	-13.02	-19.73 – -6.31	**<0.001**
ln(Mean discharge)	2.23	1.21 – 3.26	**<0.001**
Flood*	-0.57	-2.06 – 0.93	0.459
Observations	22		
R^2	0.721		

* Dummy variable added to account for outliers during two high discharge events in July and August 2021.

References

Primpke, S., Fischer, M., Lorenz, C., Gerdts, G., Scholz-Böttcher, B.M., 2020. Comparison of pyrolysis gas chromatography/mass spectrometry and hyperspectral FTIR imaging spectroscopy for the analysis of microplastics. Anal. Bioanal. Chem. 412, 8283–8298.

Simon, M., van Alst, N., Vollertsen, J., 2018. Quantification of microplastic mass and removal rates at wastewater treatment plants applying Focal Plane Array (FPA)-based Fourier Transform Infrared (FT-IR) imaging. Water Res. 142, 1–9.
